# Hospital Revenues Today and Ten Years Ago

**Published:** 1886-12-11

**Authors:** 


### Hospital Administration.

### HOSPITAL REVENUES TO-DAY AND TEN YEARS AGO.

[Continued from page 1.—Communications for this column should be addressed, "Administrator," care of the Editor of The Hospital.]

**The Influence of Hospital Sunday.**

After much consideration, and with the view of helping the hospital managers to greatly benefit by the Royal Jubilee, it has been decided to contrast the condition of the London hospitals ten years ago, *i.e.*, in the year 1876, with the state of affairs in the year 1885. Ten years is a sufficiently long interval to enable us to arrive at the true condition of affairs. Besides, such a comparison has the additional advantage of helping us to see at a glance to what extent the establishment of the Hospital Sunday Fund has affected the hospitals, beneficially or otherwise. Hospital Sunday was first systematically organised in London in the year 1876, and if the hospital revenue from charitable sources has steadily increased in the last ten years, then the institution of Hospital Sunday must be admitted to have been helpful to the hospitals individually and collectively. It may be well to explain that in the Table *charitable revenue* is made to include all receipts from voluntary sources, together with that derived from invested property, but not legacies, which are regarded as extraordinary income. In the same way *ordinary expenditure* is held to mean all outgoings each year except the sums spent to provide new, extra, and additional buildings, which are placed under the head of extraordinary expenditure.

### HOSPITAL WORK IN THE YEARS 1876 AND 1885, COMPARED.

*Table giving the population of Greater London, the number of beds, of patients, in and out, income and expenditure, with the percentages of each per* 1,000 *and* 100 *inhabitants, and per head at seventy-three London Hospitals.*

| | Year 1876. | Year 1885. | Increase per cent. in year 1885 as compared with 1876. |
|---|---|---|---|
| *Population* of Greater London .. .. | 4,286,707 | 5,199,166 | 21% |
| *No. of Beds* in 73 London Hospitals | 5,275 | 6,845 | 29% |
| Proportion of beds per 10,000 inhabitants .. .. .. | 12,34 | 13,16 | |
| *No of In-patients* | 41,448 | 58,764 | 41% |
| Percentage of In-patients to population .. .. | 971 | 1,131 | |
| *No. of Out-patients* | 525,044 | 683,815 | 27% |
| Percentage of Out-patients to population .. | 12,253 | 13,150 | |
| *Total—In- and Out-patients* .. .. .. | 566,492 | 742,579 | 28% |
| Percentage of In & Out-patients to population .. | 13.234 | 14.281 | |
| *Income* of 73 London Hospitals—Charitable .. .. | £192,736 0 0 | £256,942 0 0 | 33% |
| Amount contributed per 1,000 of population .. | 45 3 5 | 49 8 3 | |
| Do. per 100 of population .. .. | 4 10 4 | 4 18 10 | |
| Do. per head of population .. | 10,84*d.* | 11.858*d.* | |
| *Expenditure* of 73 London Hospitals | £367,848 0 0 | £457.062 0 0 | 24% |
| Total amount expended per 1,000 of population .. | 86 7 1 | 87 17 11 | |
| Do. per 100 of population .. .. | 8 12 8½ | 8 15 9½ | |
| Do. per head of population .. | 1*s.* 8.724*d.* | 1*s.* 9.095*d.* | |

**Important Points.**

At the outset, let us ascertain if there is any increased financial depression in the hospitals of London at the present time as compared with ten years ago. The really important points to establish are (1 did the public contribute less or more money to the London hospitals in 1885 than they did in 1876? and (2), is the undoubted monetary pressure which prevails at the present time due to the continuance of a state of affairs which existed to an equal, and possibly to a greater extent in 1876, than it does to-day? A careful study of the above figures will answer these questions by revealing the actual facts.

**Remarkable Figures.**

It will be seen that the London hospitals received more in charitable revenue from the people of London in 1885 than they did in 1876, which represents an increase of 33 per cent. in ten years. The expenditure, however, only increased by 24 per cent., or from £367,848 in 1876 to £457,062 in 1885. There has, therefore, been not only no falling off in the charitable income of the London hospitals during the last ten years, but an additional contribution from the public of 9 per cent. over and above the increased expenditure which the managers have had to incur to enable them to meet the additional work cast upon them by the increase in the population. It is satisfactory to see that the duty of providing for this increased work has not been shirked by the Hospital Committees. Thus, whereas the population has increased to the extent of 21 per cent. in the ten years, the hospital beds have increased by 29 per cent., *i.e.*, there are now 8 per cent. more beds available than were provided for a similar population ten years ago. It is also shown that more people in proportion used the hospitals in 1885 than in 1876, the increase in the patients amounting to 41 per cent. in the in-patient, and to 27 per cent. in the out-patient department. Putting these facts in a different way, it may be stated that in the year 1876 every 1,000 of the inhabitants of London contributed £45 to the hospitals, whilst the hospitals gave them medical relief, in return, to the value of £86 per 1,000 persons. But in 1885, whereas the hospitals gave relief to the value of £88 per 1,000, the charitable revenue they received from the public amounted to £49 per 1,000—that is, to an increased contribution of £4 5*s.* per 1,000 inhabitants. Thus the free contributions of the people to the London hospitals have increased by rather more than one penny per head per annum of the population during the last ten years, whilst the expenditure has only increased about one farthing per head. So that the charitable revenue of hospitals is three farthings per head of the population more than it was ten years ago, which represents an increase of something like £20,000 a year in the total income of the seventy-three metropolitan hospitals given in the Table. The detailed figures are too numerous to publish, but anyone may see them who cares to write us a line and make an appointment.

The One Thing Needful.

The area of public interest in all the hospitals should be extended by every possible means until the people, *as a whole*, become alive to the importance and necessity, as well as to the duty and privilege, of maintaining the hospitals in efficiency. All the hospitals should combine. The Hospitals Association offers them an excellent platform for this purpose, and by cordial co-operation they should cement and extend the desirable change in public feeling here proved to prevail, with as little delay as possible. The truth is, if the hospitals are to be kept off the rates, they really need a new departure, and the introduction of a better system of finance. Those who study the bearings of the question will become aware of the fact, that the best managed hospitals are able to provide sufficient funds to meet the increasing demands which are each year made upon their resources. What cripples them is the burden of ancient debt they have inherited from their predecessors in the management. The Metropolitan hospitals have long needed, at least, £40,000 a year more of charitable income, and until this is forthcoming, there must always be more or less financial depression. If a determined and combined effort were made to raise this additional £40,000 a year once and for all, the London hospitals would be freed at once from an intolerable burden which cripples their resources, and prevents them from doing much that they might otherwise do in the cause of suffering humanity. A capital sum of £1,000,000 will suffice to place the hospitals, once and for all, on a sound financial basis. Why should not an effort be made to raise this sum for the hospitals in commemoration of the Queen's Jubilee?

What Can and Ought to be Done.

As Lord Aberdare pointed out in *The Times* this week: "In the midst of a period of deep depression; when the difficulty of obtaining money is exceptionally great, the public has learnt with astonishment that the applications for shares in a certain Limited Liability Company have amounted, in thirty-six hours, to £126,000,000. A fact like this cannot but lead those who are interested in charitable undertakings to ask themselves whether some fraction of this unemployed capital might not be devoted to the urgent needs of our London hospitals." We venture to think it is not those who interest themselves in charitable undertakings, who ought to ask themselves this question, but rather the rich and fortunate owners of this immense capital. To start "a Jubilee Cot" for one hospital, however excellent an idea in itself, shows so lamentable an absence of a proper sense of the opportunity and the occasion, as to border on the ridiculous. Even the endowment of a Jubilee Bed in every hospital throughout the British Empire would be an inadequate proposal, whilst a combined effort by all the Metropolitan hospitals to promote a scheme which will establish them, once and for all, on a sound basis by raising £1,000,000, would be worthy of our day and generation, and prove the most memorable incident in the reign of our beloved Queen.

